# Beyond Infection and Lupus Flare: MPO‐ANCA Microscopic Polyangiitis Causing Diffuse Alveolar Hemorrhage in a Patient With Systemic Lupus Erythematosus

**DOI:** 10.1002/ccr3.71621

**Published:** 2025-12-14

**Authors:** Abu Bocus, Tamer Mohamed Zaalouk, Partha Chowdhury, Siraj Nasim

**Affiliations:** ^1^ Acute Medicine Department Queen Elizabeth The Queen Mother Hospital Margate UK

**Keywords:** ANCA‐associated vasculitis, diffuse alveolar hemorrhage, microscopic polyangiitis, MPO‐ANCA, systemic lupus erythematosus

## Abstract

We describe an elderly woman with systemic lupus erythematosus (SLE) who presented with dyspnoea, anemia, and bilateral pulmonary infiltrates initially diagnosed as community‐acquired pneumonia. Despite appropriate antibiotics, she failed to improve, and all microbiological investigations remained negative. Further immunological evaluation revealed perinuclear antineutrophil cytoplasmic antibody (p‐ANCA) with anti‐myeloperoxidase (MPO) specificity, establishing a diagnosis of microscopic polyangiitis (MPA) complicated by diffuse alveolar hemorrhage (DAH). This case illustrates a complex diagnostic dilemma in which pulmonary hemorrhage in a patient with SLE may represent either lupus‐related pulmonary vasculitis or a new ANCA‐associated process. It highlights the importance of reconsidering the diagnosis in non‐resolving respiratory disease, particularly in patients with overlapping autoimmune conditions.

## Key Clinical Message

In patients with systemic lupus erythematosus who develop new pulmonary infiltrates that fail to respond to antibiotics, diffuse alveolar hemorrhage secondary to microscopic polyangiitis should be strongly considered. Early integration of serological markers, targeted CT imaging, and simple bedside tests such as urinalysis is crucial to distinguish vasculitis from a lupus flare. Timely recognition and prompt immunosuppressive therapy can be lifesaving and prevent permanent pulmonary damage.

## Graphical abstract

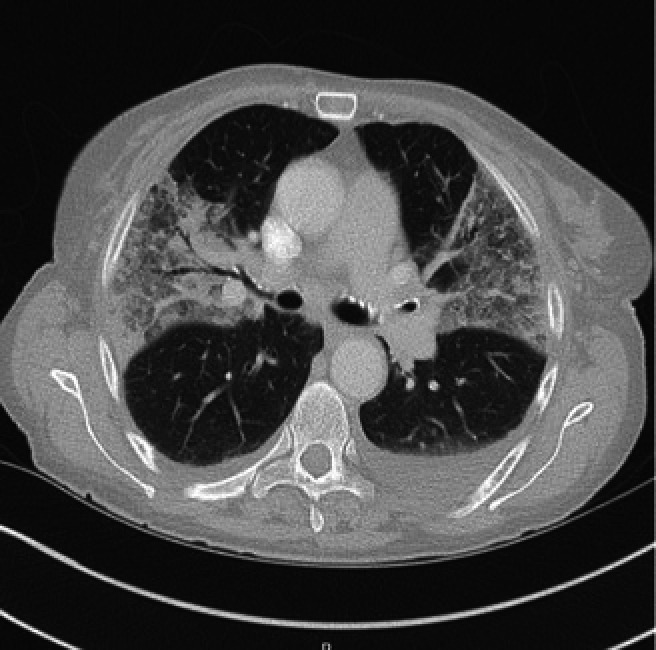

## 1 Introduction

Microscopic polyangiitis (MPA) is an antineutrophil cytoplasmic antibody (ANCA)–associated small‐vessel vasculitis with frequent pulmonary and renal involvement. Diffuse alveolar hemorrhage (DAH) is a potentially life‐threatening manifestation that may present with dyspnoea, anemia, haemoptysis, and bilateral pulmonary infiltrates, often mimicking pneumonia or pulmonary oedema [1, 2]. Systemic lupus erythematosus (SLE) can produce a similar pulmonary picture through immune complex–mediated capillaritis. Because clinical and radiological features overlap significantly, distinguishing MPA‐related DAH from SLE‐associated DAH can be challenging, particularly when patients have dual autoimmune diagnoses or atypical serological profiles. Failure to recognize an underlying ANCA‐associated vasculitis in a patient with known SLE may delay appropriate immunosuppressive therapy and increase morbidity. This case highlights the diagnostic complexity of evaluating DAH in an SLE patient and underscores the need for a high index of suspicion for overlapping vasculitis processes.

## 2 Case Presentation

A woman in her 70s with longstanding SLE, primary biliary cirrhosis, hypothyroidism, portal hypertension, and osteoarthritis presented with a 3‐week history of progressive dyspnoea and fatigue. Initial investigations revealed severe anemia (hemoglobin 54 g/L) with iron‐deficiency indices and no overt bleeding. She received three units of red blood cells and was discharged with outpatient follow‐up arranged.

**FIGURE 1:**
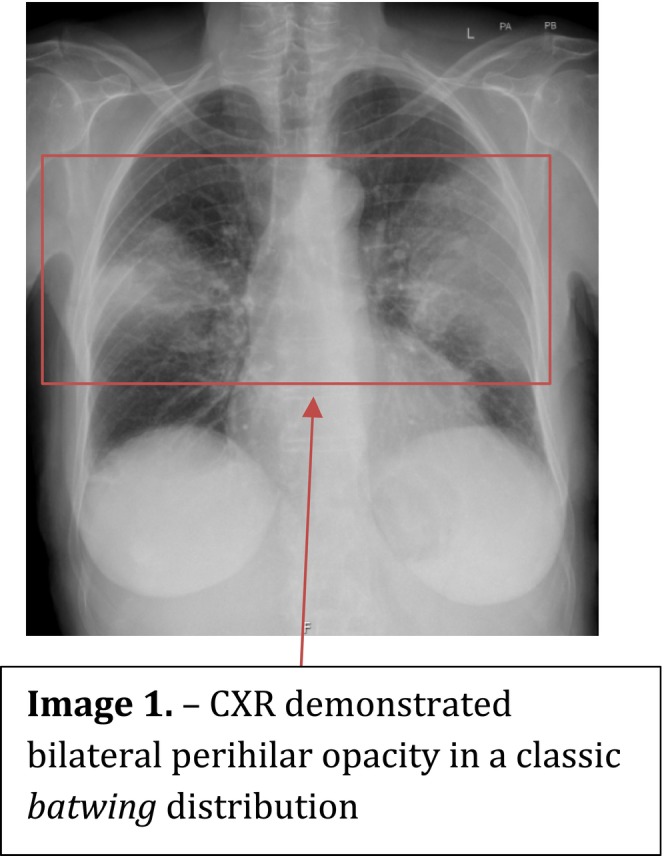
CXR demonstrated bilateral perihilar opacity in a classic *batwing* distribution.

Five days later, she represented with fever (38.4°C), productive cough, chest discomfort, and worsening breathlessness. On examination, she was tachycardic (106 bpm), hypotensive (104/45 mmHg), and had diffuse bilateral crackles on chest auscultation. ECG showed sinus rhythm with occasional ventricular ectopy. Chest radiography demonstrated bilateral perihilar “batwing” opacities (Figure 1), suggesting pulmonary oedema or hemorrhage. Laboratory testing (Table 1) showed a white cell count of 14.0 × 10^9^/L, C‐reactive protein (CRP) 120 mg/L, and preserved renal function (creatinine 82 μmol/L; eGFR 72 mL/min). Bedside urinalysis revealed large blood with negative leukocytes, protein, and nitrites, prompting further evaluation.

She was initially treated for community‐acquired pneumonia with clarithromycin and vancomycin. Sputum cultures, viral PCR panels, and atypical pneumonia screens were negative. After 48 h of antibiotic therapy, she failed to improve clinically and developed increasing oxygen requirements. Repeat inflammatory markers remained elevated, leading to a vasculitis screen and CT chest.

**FIGURE 2:**
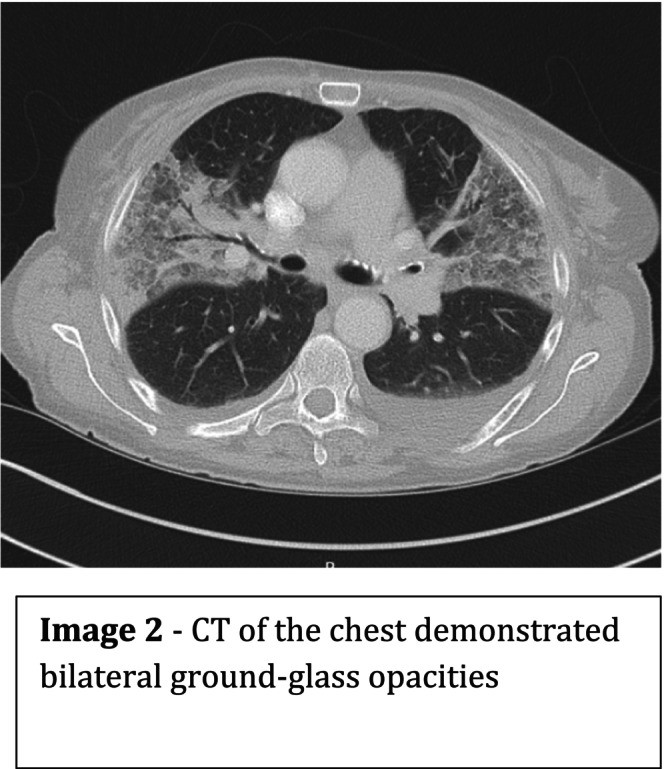
CT of the chest demonstrated bilateral ground‐glass opacities.

CT imaging (Figure 2) demonstrated bilateral, predominantly upper‐lobe ground‐glass opacities consistent with diffuse alveolar hemorrhage (DAH). Immunological testing showed negative ANA and anti‐dsDNA, normal complement (C3 and C4) levels, but strongly positive p‐ANCA with anti‐MPO titer. The presence of MPO‐ANCA positivity in a patient with background SLE raised the possibility of an overlapping vasculitis process rather than a lupus flare.

Given the imaging findings, serological profile, and lack of infective evidence, a working diagnosis of MPA with DAH was made. Bronchoscopy subsequently confirmed alveolar hemorrhage with haemosiderin‐laden macrophages.

**FIGURE 3:**
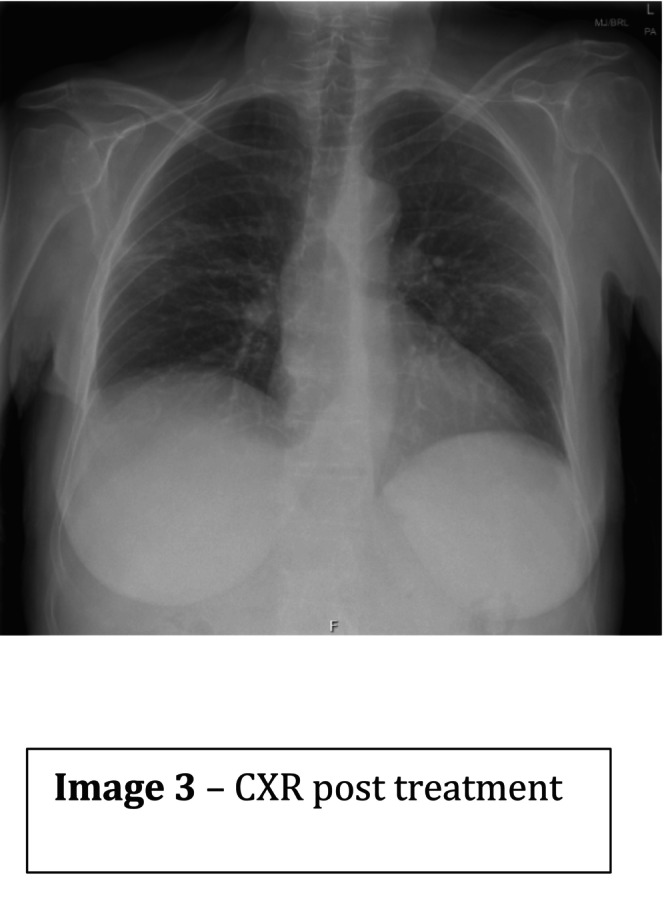
CXR post treatment.

**FIGURE 4:**
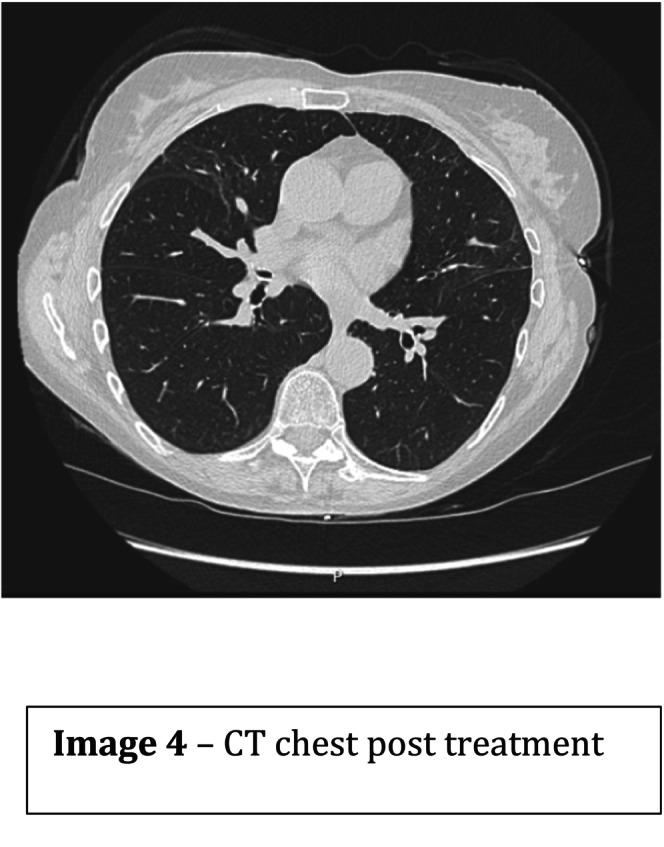
CT chest post treatment.

High‐dose intravenous methylprednisolone (500 mg daily for 3 days) was commenced, followed by a tapering oral prednisolone regimen in line with the PEXIVAS protocol. Rituximab was selected for induction therapy instead of cyclophosphamide due to underlying chronic liver disease (primary biliary cirrhosis). She also received bone protection, proton pump inhibitor prophylaxis, and co‐trimoxazole for Pneumocystis jirovecii prophylaxis. The patient improved rapidly, with resolution of dyspnoea, normalization of inflammatory markers, and marked radiological improvement over subsequent weeks (Figures 3 and 4). She was referred for ongoing rheumatology follow‐up and long‐term vasculitis surveillance [3].

Differential diagnosis

– Community‐acquired pneumonia
– Cardiogenic pulmonary oedema
– Pulmonary alveolar proteinosis
– Pneumocystis jirovecii pneumonia
– SLE‐induced diffuse alveolar hemorrhage
– Pulmonary vasculitis‐related hemorrhage (final diagnosis)

## 3 Investigations

See Figures 1, 2, 3, 4, Table 1.

**TABLE 1:** Summary of laboratory and diagnostic investigations.

Investigation	Result	Reference range	Interpretation/Comment
Hemoglobin (Hb)	81 g/L	120–160 g/L	Severe anemia
White cell count (WBC)	14.0 × 10^9^/L	4.0–11.0 × 10^9^/L	Mild leukocytosis
Platelets	312 × 10^9^/L	150–400 × 10^9^/L	Normal
C‐reactive protein (CRP)	120 mg/L	< 5 mg/L	Markedly elevated
Erythrocyte sedimentation rate (ESR)	90 mm/h	< 20 mm/h	Elevated
Urinalysis (dipstick)	Large blood	Negative	Microscopic hematuria without infection
ANA	Negative	Negative	No active lupus flare
Anti‐dsDNA antibody	Negative	Negative	No active lupus flare
Complement C3	0.78 g/L (low‐normal)	0.75–1.65 g/L	Mild reduction
Complement C4	Normal	0.14–0.54 g/L	Normal
p‐ANCA (anti‐MPO)	Positive	—	
Chest radiograph	Bilateral bat‐wing opacities	—	
CT chest	Upper‐lobe ground‐glass opacities	—	Consistent with diffuse alveolar hemorrhage
Bronchoalveolar lavage	Positive	—	Confirms alveolar hemorrhage

Abbreviations: ANA, antinuclear antibody; CRP, C‐reactive protein; eGFR, estimated glomerular filtration rate; ESR, erythrocyte sedimentation rate; Hb, hemoglobin; MPA, microscopic polyangiitis; MPO, myeloperoxidase; WBC, white blood cells.

## 4 Discussion

Diffuse alveolar hemorrhage (DAH) is a fulminant pulmonary emergency most commonly associated with ANCA‐associated vasculitis (AAV) and systemic lupus erythematosus (SLE). Distinguishing between these aetiologies is particularly challenging because the clinical, radiological, and laboratory features frequently overlap. Both conditions may present with acute dyspnoea, falling hemoglobin, hypoxaemia, and bilateral ground‐glass opacities, making early diagnosis dependent on careful integration of serological markers, renal findings, and the clinical trajectory. In patients with established SLE, new pulmonary infiltrates are often attributed to infection or lupus pneumonitis, a diagnostic assumption that may delay recognition of an alternative coexisting vasculitis process such as microscopic polyangiitis (MPA). This case exemplifies the diagnostic uncertainty that arises when overlapping autoimmune disorders coexist and underscores the need for early reevaluation of non‐resolving respiratory disease.

The reported incidence of DAH in SLE ranges from 2% to 5%, whereas up to 20%–30% of patients with AAV—particularly MPA—develop DAH at some point in their disease course [1, 2]. The underlying immunopathogenesis differs markedly: SLE‐associated DAH is typically driven by immune complex deposition with complement consumption, reflected in high anti‐dsDNA titres and low C3/C4 levels, while MPA‐related DAH results from pauci‐immune capillaritis triggered by MPO‐ANCA–mediated neutrophil activation. In our patient, the complete absence of lupus serological activity—negative ANA, negative dsDNA, and normal complement levels—made an SLE flare biologically unlikely. In contrast, the strongly positive MPO‐ANCA level provided compelling evidence of an AAV process. Several studies have highlighted that MPO‐ANCA positivity in patients with SLE strongly correlates with the presence of an overlapping vasculitic syndrome rather than atypical lupus activity, with reported overlap rates between 5% and 10% [4, 5].

Renal involvement further helped differentiate the underlying pathology. Although glomerulonephritis is a hallmark of MPA, between 10% and 15% of patients may present initially with isolated pulmonary disease, delaying recognition of vasculitis [3]. Our patient exhibited microscopic haematuria without proteinuria or cellular casts and maintained normal renal function—findings compatible with early or lung‐limited MPA. In contrast, DAH in SLE is frequently associated with active lupus nephritis and significant complement consumption. The absence of these typical lupus features, in the context of new MPO‐ANCA positivity, made MPA the more plausible diagnosis. This supports recent literature emphasizing the value of combined serological and renal assessment in the evaluation of DAH in autoimmune disease [6].

The patient's initial presentation was misinterpreted as community‐acquired pneumonia, reflecting a common diagnostic pitfall. Bilateral ground‐glass opacities and raised inflammatory markers often prompt empirical antibiotic therapy, and anchoring bias may reinforce an infectious diagnosis even when the clinical course is inconsistent. A growing body of evidence highlights the importance of reassessing the diagnosis after 48–72 h of persistent symptoms and negative microbiology, particularly in patients with autoimmune backgrounds [7]. Early CT imaging is strongly recommended in non‐resolving pneumonia because characteristic patterns—such as upper‐lobe predominant ground‐glass opacities—should prompt consideration of DAH and urgent immunological evaluation.

Management of MPA‐related DAH requires rapid initiation of high‐dose glucocorticoids and an induction agent to prevent respiratory failure and multiorgan involvement. Both cyclophosphamide and rituximab are accepted first‐line induction therapies, with comparable efficacy demonstrated in the RAVE and RITUXVAS trials [8, 9]. Rituximab offers particular advantages in older patients and those with significant comorbidities. In this case, rituximab was chosen due to primary biliary cirrhosis and the associated risk of cyclophosphamide toxicity. The patient's rapid clinical and radiological improvement following methylprednisolone and rituximab supports the accuracy of the working diagnosis and further differentiates MPA from SLE, as lupus‐associated DAH often requires intensified lupus‐directed therapy or plasmapheresis.

This case contributes to the expanding literature on SLE–AAV overlap syndromes, which remain rare but clinically significant. Overlap cases frequently present with atypical serological patterns, diagnostic ambiguity, and a tendency toward more severe pulmonary manifestations. Recognizing such overlap is crucial because misattributing symptoms to a lupus flare alone may result in delayed initiation of ANCA‐directed therapy, which is essential for preventing irreversible lung injury. Current recommendations emphasize maintaining a high index of suspicion for vasculitis in SLE patients with DAH when the serological profile does not align with classic lupus activity or when the clinical course deviates from expected recovery patterns [10].

## Author Contributions

**Abu Bocus:** conceptualization, data curation, investigation, methodology, writing – original draft. **Tamer Mohamed Zaalouk:** formal analysis, supervision, writing – review and editing. **Partha Chowdhury:** investigation, writing – review and editing. **Siraj Nasim:** writing – review and editing.

## Funding

The authors have nothing to report. The research was performed as part of the employment of the authors in QEQM hospital, Margate (EKUHFT).

## Consent

Written informed consent was obtained from the patient for publication of this case report.

## Conflicts of Interest

The authors declare no conflicts of interest.

## Data Availability

This case report does not include any publicly available datasets. All clinical data referenced were obtained from routine patient care and have been fully anonymized to protect patient confidentiality.
